# Assessment of information quality in the largest Danish Facebook group on atopic diseases: a mixed bag of help and harm

**DOI:** 10.3389/falgy.2024.1378383

**Published:** 2025-01-10

**Authors:** Simon Høj, Simon Francis Thomsen, Hanieh Meteran, Torben Sigsgaard, Howraman Meteran

**Affiliations:** ^1^ Department of Dermatology, Venereology, and Wound Healing Centre, Copenhagen University Hospital-Bispebjerg, Copenhagen, Denmark; ^2^ Department of Public Health, Environment, Occupation, and Health, Aarhus University, Aarhus, Denmark; ^3^ Department of Biomedical Sciences, University of Copenhagen, Copenhagen, Denmark; ^4^ Department of Endocrinology, Copenhagen University Hospital-Hvidovre, Hvidovre, Denmark; ^5^ Department of Respiratory Medicine, Copenhagen University Hospital-Hvidovre, Hvidovre, Denmark; ^6^ Department of Respiratory Medicine, Zealand University Hospital, Roskilde—Næstved, Næstved, Denmark

**Keywords:** asthma, atopic diseases, dermatitis, rhinitis, social media

## Abstract

**Background:**

Patient education is an important part of the management of atopic diseases such as allergic rhinitis, atopic dermatitis, and asthma. Given the increasing reliance on social media platforms such as Facebook for health-related discourse, there are concerns about the accuracy and quality of the shared information.

**Aim:**

The aim of this study was to categorize and assess the quality of the information shared within the largest Danish Facebook group focusing on atopic diseases.

**Method:**

A total of 652 posts and 7,515 comments were scrutinized, classifying each as useful, misleading, or neutral.

**Results:**

The analysis predominantly identified discussions around asthma (40%), allergic rhinitis (21%), and eczema (5%), with the majority of queries posed by women and related to symptoms and medications. The results indicated that 11% of comments were deemed useful, whereas 12% were categorized as misleading, with the bulk of comments being neutral. Concerningly, 52% of the comments promoting behavioral change were found to be misleading.

**Conclusion:**

Although the Facebook group serves as a hub for peer support, its utility as a reliable educational resource is compromised. Overall, 12% of the comments were classified as misleading, while more than half of the advice encouraging behavioral change was misleading.

## Introduction

Atopic diseases are common chronic conditions that affect a significant portion of the global population. Asthma alone affects more than 300 million people worldwide, and the prevalence of allergic rhinitis is still increasing and affects approximately 30% of the global population (1, 2). Atopic dermatitis affects between 15%–20% of children and 1%–3% of adults (3). Atopic diseases are uncurable and require lifelong treatment and access to useful patient information to optimally manage the diseases.

Facebook has become increasingly popular as a platform for patients to seek health-related information and share personal experiences with others with similar conditions. Due to the lack of peer-reviewed content and the platform's open accessibility for posting questions and comments, the information shared on the platform may vary in quality and accuracy. Previous studies have shown that a large proportion of the most viewed content on atopic diseases on other online platforms is misleading (4–6).

Facebook's algorithm prioritizes content based on relevance and engagement, known as a relevancy score. This rating system considers several factors, including user engagement measures such as likes, comments, and shares and the duration of time spent interacting with the posts (7). Posts with higher scores are likelier to be featured at the top of the newsfeed, with notifications specifically addressed to other group members. Despite its effectiveness at increasing interaction, there remain potential risks associated with the algorithm since misleading content may still receive high levels of endorsement despite being inaccurate (8). This study aimed to examine the topics with the most frequently asked questions and the quality of the responses within Facebook groups on atopic diseases.

## Materials and methods

The largest (based on number of members) and most active (based on daily posts) Danish Facebook group regarding atopic diseases was identified. There were seven groups with more than 500 members, including the group we examined. Among these groups, there was also a group on milk allergy, which was not restricted to true allergy but also lactose intolerance, a group only focusing on food allergy, and one group focusing on eczema. Using a number generator, two random weeks were chosen for each month in 2022, where posts and comments were screened, recorded, and analyzed by two authors (SH and HoM). A total of 652 posts and 7,515 comments were collected for further analyses, and the following data were obtained for each post: the date of release, likes, the sex of the poster (assessed by the name), and comments. In addition, all the posts were categorized by topic, and both posts and comments were assessed based on quality, as detailed below.

Two authors (SH and HoM) assessed the quality and content of the post and comments, and any disagreements were resolved through consensus among all authors, with a high agreement with a Cohen's kappa of 0.89. The content was classified into three categories: (1) useful: the post/comment conveys scientifically correct information; (2) misleading: the post/comment conveys at least one scientifically wrong or unproven detail; or (3) neither useful nor misleading, post/comment that is not misleading but does not provide useful information. Posts/comments that were categorized as not covering descriptive content did not encourage any type of behavioral change, ranging from peer support to incomprehensible indifferences. Previous studies have used this categorization to determine the quality of content on social media (4–6).

Descriptive statistics were used for the categorization of the topic of the post, and Fischer’s exact test or chi-squared test was used based on the expected frequency. *P*-values were calculated to test for the difference in the proportion of useful vs. misleading comments.

## Results

Of the 652 posts, 40% were about asthma, 21% about allergic rhinitis, 5% about eczema, and 34% were not about a specific disease but, e.g., about symptoms or medication at a general level. In total, 89% of the posts were questions and primarily authored by women, who contributed 84% of the total posts.

The most frequently asked questions were about symptoms (56%), followed by questions about medication (14%), with 10% and 4% of the latter pertaining to inhalers and tablets, respectively. In addition, 79% of the posts were self-inquiries, while 21% were written on behalf of a relative.

Of the 7,515 comments, 11% were classified as useful, 12% contained misleading information, and 77% neither contributed useful information nor conveyed misinformation.

The misleading comments were distributed across the topics: asthma 36%; allergic rhinitis 24%; eczema 13%; and non-disease specific 26%.

Among the 1,698 comments (23% of total comments) encouraging behavioral change, more than half (52%) provided inaccurate information. Comments about exercise had the highest percentage of misleading content with 21%, followed closely by comments about nutrition with 15%. Statistically significant differences were observed between useful information and misinformation in three categories, namely prevention, lung function, and miscellaneous, with a higher proportion of useful information being reported (shown in Table 1).

**Table 1:** Comment characteristics according to their usefulness category.

**Topic in the comments**	**Useful *N* (%)**	**Misleading *N* (%)**	**Neither *N* (%)**	***P*-value**
				**Useful vs. misleading**
Medication	151 (14%)	128 (12%)	811 (74%)	0.14
Prevention	30 (18%)	11 (7%)	128 (76%)	<0.01
Risk factors	4 (13%)	3 (10%)	24 (78%)	0.69
Diagnosis	47 (11%)	31 (7%)	356 (82%)	0.06
Alternative therapy	5 (7%)	8 (11%)	62 (83%)	0.39
Criticism of healthcare system	19 (13%)	18 (12%)	107 (74%)	0.86
Miscellaneous	45 (7%)	23 (4%)	570 (89%)	<0.01
Exercise	11 (15%)	15 (21%)	47 (64%)	0.39
Nutrition	31 (21%)	22 (15%)	91 (63%)	0.17
Smoking	15 (23%)	8 (13%)	41 (64%)	0.11
Lung function	21 (37%)	4 (7%)	32 (57%)	<0.01
Biologics	2 (13%)	2 (13%)	11 (74%)	1.0
Symptoms	509 (11%)	559 (12%)	3,531 (77%)	0.11

“Miscellaneous” covers posts such as peer support, politics, events, questions regarding the availability or procurement of specific products, and other things that do not fit into the other categories.

## Discussion

To our knowledge, this is the first study analyzing the quality of content within Facebook groups about atopic diseases. Earlier studies have found substantial misinformation on other social media platforms such as YouTube (4–6), but Facebook differs by providing the possibility of higher user engagement among people with the same disease. This study reveals that the largest Danish Facebook group focusing on atopic diseases is highly active in post and comment activity. Interestingly, we observed that a high proportion (>75%) of the questions were asked by women across all topics, except for posts related to alternative treatment (60%). This sex ratio does not reflect the prevalence of atopic diseases according to sex and needs further investigation. The majority of comments were aimed at providing peer support and sharing personal experiences. However, our findings demonstrate a noteworthy prevalence of misleading information within the comments aimed at fostering behavioral change. These results suggest that although patients with atopic diseases can gain useful information in disease-specific Facebook groups, the overall utility is questionable. Due to the study only focusing on Danish Facebook users, we suggest similar studies be conducted to investigate whether the same pattern emerges in other countries. A study evaluating 500 posts on dental caries on Facebook found that 41% of the posts were misinformative (9). A systematic review showed that the level of misinformation depends on the specific social media platform, the specific disease, and the specific topic within a given disease (10). We recommend that health professionals consider allocating resources to accommodate the demand for peer support, reflection, and answers, which are not fulfilled within clinical practice. Health providers should be aware of the patient's need for more interaction and be able to guide patients toward evidence-based information on atopic diseases. Initiatives focused on addressing unmet needs in patient education for atopic diseases could gain valuable insights by utilizing disease-specific Facebook groups to pinpoint the most pertinent topics. Finally, the role of social media algorithms as part of a solution to combat misinformation cannot be underestimated and we have previously seen that significant interventions have been made by specific platforms (11).

## Conclusion

The largest Danish Facebook group focusing on atopic diseases offers active engagement and peer support, but its comments contain a significant amount of misleading information encouraging behavioral change among peers. Healthcare providers could use insights from disease-specific Facebook groups to effectively address unmet needs in patient education.

## Data Availability

The datasets presented in this study can be found in online repositories. The names of the repository/repositories and accession number(s) can be found here: we used data from Facebook.
